# Can the Cancer and Leukemia Group B 140503 trial support choice of segmental versus wedge resection for non–small cell lung cancer?

**DOI:** 10.1016/j.xjon.2026.101874

**Published:** 2026-05-28

**Authors:** Benjamin A.Y. Cher, Qinyun Lin, Kenneth A. Frank, James D. Maloney, Courtney J. Balentine, Andrea L. Axtell

**Affiliations:** a Wisconsin Surgical Outcomes Research Program (WiSOR), Department of Surgery, School of Medicine and Public Health, University of Wisconsin-Madison, Madison, Wis; b School of Public Health and Community Medicine, University of Gothenburg, Gothenburg, Sweden; c College of Education, Michigan State University, East Lansing, Mich; d Division of Thoracic Surgery, Department of Surgery, School of Medicine and Public Health, University of Wisconsin-Madison, Madison, Wis

**Figure:**
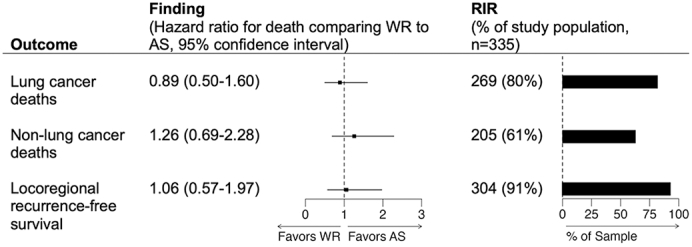
Assessment of the comparison using the robustness of inferences to replacement (RIR).

**Central Message**

Analysis using the robustness of inferences to replacement found that the findings of the post-hoc analysis of CALGB 140503, comparing wedge resection versus anatomic segmentectomy, were robust.

The optimal extent of surgical resection for clinical T1aN0 non–small cell lung cancer ≤2 cm is highly controversial.^1^ The Cancer and Leukemia Group B 140503 trial has supported parenchymal-sparing sublobar resection compared with lobar resection.^2^ However, the trial included both anatomic segmentectomy (AS) and wedge resection (WR) in the sublobar treatment group, and the primary analysis did not differentiate between these 2 approaches. The most oncologically appropriate sublobar resection remains unclear and is affected by many factors, including tumor histology and patient characteristics.^1^

The authors of the original trial published a post-hoc analysis, which found no significant survival difference between WR and AS.^3^ However, many thoracic surgeons question these post-hoc results because of concerns about unmeasured confounding, selection bias, and sample size.^1^ There remains wide variation in practice patterns.^4^ Debates about this topic are often irreconcilable because thoracic surgeons and patients lack the necessary tools to understand whether the post-hoc findings are strong enough to drive practice change.

In this context, we assessed the robustness of the findings of the post-hoc analysis using a novel method for sensitivity analysis called the robustness of inferences to replacement (RIR). The RIR quantifies the strength of the findings against the aforementioned concerns by measuring how resistant the findings are to hypothetical changes in patient outcomes.^5^ The RIR has a more direct clinical interpretation than standard statistics such as *P* values and 95% CIs because it measures robustness in terms of how many patients in a study would have had to experience different outcomes—or be “replaced” with hypothetical patients who experienced different outcomes—for the study findings to be reversed. Our goal was to understand whether the post-hoc analysis was sufficiently robust to guide clinical decision-making.

## Methods

We applied the RIR to the principal findings of the post-hoc analysis. The RIR quantifies how much resampling from a null-hypothesis population would be necessary to change the study findings such that a nonsignificant finding becomes statistically significant.^5^

For a study finding no significant survival difference between WR and AS, the RIR quantifies how many study patients would need to be replaced by patients with the threshold survival difference (minimum smallest statistically significant difference) for the result to be changed so there is a significant difference in survival between WR and AS. If the RIR is high, then a study is considered more robust. It is unlikely that controlling for unmeasured variables or repeating the study with a new sample would generate a different result.

Approval by the institutional review board was not necessary for this study of publicly available published data. Consent was not required.

## Results

**Figure 1:**
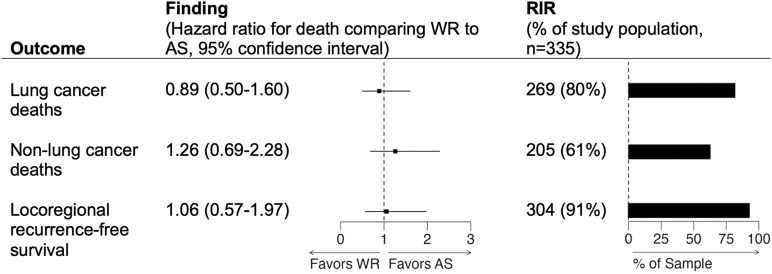
Assessment of the robustness of the findings of the post-hoc comparison between anatomic segmentectomy (*AS*, n = 131) and wedge resection (*WR*, n = 204) for patients with clinical T1aN0 non–small cell lung cancer using the robustness of inferences to replacement (*RIR*).

Using a post-hoc analysis, we found no significant difference in lung cancer deaths (hazard ratio [HR] for death 0.89; 95% CI 0.50-1.60) between WR (n = 204) and AS (n = 131), Figure 1. The RIR for this finding was 269 (of 335), meaning that 80.2% of the study sample, for which there was no survival difference, would need to be replaced with patients with the threshold survival difference for the result to change, so that there was a significant difference in survival between WR and AS.

The post-hoc analysis also found no significant difference in risk of nonlung cancer deaths (HR, 1.26; 95% CI, 0.69-2.28) between WR and AS. The RIR for this finding was 205, meaning 61.2% of the study sample for which there was no survival difference would need to be replaced in a similar fashion for the result to change.

In addition, there was no difference in locoregional recurrence-free survival (HR, 1.06; 95% CI, 0.57-1.97) between WR and AS. The RIR for this finding was 304, meaning 90.6% of the sample for which there was no survival difference would need to be replaced for the result to change.

## Discussion

The post-hoc analysis's findings of no survival differences between AS and WR are robust, because the sensitivity analysis found that 60 to 90% of patients would have needed to experience different outcomes for the results to change. This suggests that a new study directly comparing AS versus WR would likely come to the same conclusion, even with better control for confounders, different selection criteria, and/or a larger sample size. It is unlikely there is a survival difference between AS and WR for patients with T1aN0 non–small cell lung cancer ≤2 cm.

## Conflict of Interest Statement

The authors reported no conflicts of interest.

The *Journal* policy requires editors and reviewers to disclose conflicts of interest and to decline handling or reviewing manuscripts for which they may have a conflict of interest. The editors and reviewers of this article have no conflicts of interest.

## References

[1] Ceccarelli I., Durand M., Seguin-Givelet A. (2025). The evolving role of wedge resection in early-stage non–small cell lung cancer: a literature review. Transl Lung Cancer Res.

[2] Altorki N., Wang X., Kozono D. (2023). Lobar or sublobar resection for peripheral stage IA non–small-cell lung cancer. N Engl J Med.

[3] Altorki N., Wang X., Damman B. (2024). Lobectomy, segmentectomy, or wedge resection for peripheral clinical T1aN0 non–small cell lung cancer: a post hoc analysis of CALGB 140503 (Alliance). J Thorac Cardiovasc Surg.

[4] Seder C.W., Chang S.C., Towe C.W. (2025). Anatomic lung resection is associated with improved survival compared with wedge resection for stage IA (≤2 cm) NSCLC. J Thorac Oncol.

[5] Frank K.A., Lin Q., Maroulis S. (2021). Hypothetical case replacement can be used to quantify the robustness of trial results. J Clin Epidemiol.

